# Neuropsychological and neurophysiological benefits from white noise in children with and without ADHD

**DOI:** 10.1186/s12993-016-0095-y

**Published:** 2016-03-15

**Authors:** Simon Baijot, Hichem Slama, Göran Söderlund, Bernard Dan, Paul Deltenre, Cécile Colin, Nicolas Deconinck

**Affiliations:** Center for Research in Cognition and Neurosciences (CRCN), Université Libre de Bruxelles (ULB), Campus du Solbosch CP 191, Avenue F.D. Roosevelt 50, CP 151, 1050 Brussels, Belgium; Research Unit in Cognitive Neurosciences (UNESCOG), Université Libre de Bruxelles (ULB), 1050 Brussels, Belgium; Department of Neurology, Queen Fabiola Children’s University Hospital (HUDERF), Université Libre de Bruxelles (ULB), Avenue Jean-Joseph Crocq, 15, 1020 Brussels, Belgium; Laboratory of Cognitive and Sensory Neurophysiology, CHU Brugmann, Université Libre de Bruxelles (ULB), Place Van Gehuchten, 4, 1020 Brussels, Belgium; Neuropsychology and Functional Neuroimaging Research Group (UR2NF), Université Libre de Bruxelles (ULB), 1050 Brussels, Belgium; Department of Clinical and Cognitive Neuropsychology, Erasme Hospital, Université Libre de Bruxelles (ULB), Route de Lennik, 808, 1070 Brussels, Belgium; Faculty of Teacher Education and Sports, Sogn og Fjordane, University College, Sogndal, Norway; Inkendaal Rehabilitation Hospital, Vlezenbeek, Belgium

**Keywords:** ADHD, White noise, ERP (P300), Dopamine, Optimal stimulation

## Abstract

**Background:**

Optimal stimulation theory and moderate brain arousal (MBA) model hypothesize that extra-task stimulation (e.g. white noise) could improve cognitive functions of children with attention-deficit/hyperactivity disorder (ADHD). We investigate benefits of white noise on attention and inhibition in children with and without ADHD (7–12 years old), both at behavioral and at neurophysiological levels.

**Methods:**

Thirty children with and without ADHD performed a visual cued Go/Nogo task in two conditions (white noise or no-noise exposure), in which behavioral and P300 (mean amplitudes) data were analyzed. Spontaneous eye-blink rates were also recorded and participants went through neuropsychological assessment. Two separate analyses were conducted with each child separately assigned into two groups (1) ADHD or typically developing children (TDC), and (2) noise beneficiaries or non-beneficiaries according to the observed performance during the experiment. This latest categorization, based on a new index we called “Noise Benefits Index” (NBI), was proposed to determine a neuropsychological profile positively sensitive to noise.

**Results:**

Noise exposure reduced omission rate in children with ADHD, who were no longer different from TDC. Eye-blink rate was higher in children with ADHD but was not modulated by white noise. NBI indicated a significant relationship between ADHD and noise benefit. Strong correlations were observed between noise benefit and neuropsychological weaknesses in vigilance and inhibition. Participants who benefited from noise had an increased Go P300 in the noise condition.

**Conclusion:**

The improvement of children with ADHD with white noise supports both optimal stimulation theory and MBA model. However, eye-blink rate results question the dopaminergic hypothesis in the latter. The NBI evidenced a profile positively sensitive to noise, related with ADHD, and associated with weaker cognitive control.

## Background

Attention-deficit/hyperactivity disorder (ADHD) is a highly prevalent developmental disorder that affects about 5 % of school-aged children and adolescents [1–4]. These children typically exhibit pervasive behavioral symptoms of hyperactivity, inattention and impulsivity [1], which substantially affect their quality of life (for a review, see [5]). Moreover, these symptoms are associated with adverse educational [6], interpersonal [7], and occupational outcomes [8]. Furthermore, deficits in attention, cognitive and executive functioning are considered as core behavioral symptoms in ADHD and are concerned in most contemporary ADHD models [9]. The most prominent deficits seen in ADHD are response inhibition [10], inattention (vigilance), working memory, planning [11] and reaction time (RT), particularly RT variability [12].

While stimulant medication has been shown to improve behavioral symptoms [13] and school performance in ADHD [14], adverse effects from such medication have also been reported [15–19]. The immediate environment, which is the main concern of the present study, is also known to have a significant impact on the expression of certain ADHD symptoms such as hyperactivity, impulsivity or inattention [20–26].

Several models, including the optimal stimulation theory [20], the cognitive-energetic model [27], the stochastic resonance (SR) effect [28] and the moderate brain arousal (MBA) model [29], have tentatively incorporated an improvement of cognitive functioning related to environmental stimulation. The optimal stimulation theory is based on a homeostatic model, suggesting that each individual has its own biologically determined optimal level of arousal enabling him/her to reach the best level of cognitive functioning [20, 30]. Zentall, Zentall [30] hypothesized that children with ADHD suffer from under-arousal, which lowers their level of performance under “normal” conditions. They interpreted the restless and inattentive behavior of ADHD children as self-stimulation in order to raise their arousal level and, consequently, performance. Zentall, Zentall [30] suggest that the motor activity of children with ADHD increases more when they are exposed to a stimulus-poor environment in order to reach their high-stimulation threshold. The optimal stimulation theory was first proposed as a theoretical model but was later supported by empirical behavioral evidence from improvement in children with ADHD when extra-task stimulation was added, such as background linguistic noise during a reading/arithmetic task [31], pictures during a continuous performance test (CPT) auditory task [32], colored items during a CPT task [33, 34] and background music during arithmetic tasks [35]. Thus, apparent distraction might have a positive effect on performance and is therefore not always detrimental. In line with the optimal stimulation theory, the cognitive-energetic model postulates that ADHD symptoms and deficits occur because of problems with regulating energetic factors [27]. Sergeant et al. [27] suggest that performance is not only influenced by cognitive capacity but also by environmentally determined levels of arousal and activation as well as the extent to which variations in these energetic factors can be managed to ensure optimal performance.

One possible explanation for why adding stimulation might be beneficial lies in the stochastic resonance (SR) phenomenon [36]. SR is a phenomenon in which an optimal amount of random noise (e.g. white noise^1^), may be beneficial for cognitive performance under certain circumstances [36]. Jepma et al. [37] showed, for example, that task-irrelevant auditory white noise can speed up responses to stimuli in the visual modality.

The MBA model [38] is a neurocomputational model related to the concept of SR but has been developed in the framework of ADHD research. MBA model posits that random noise in the environment introduces, through the perceptual system, internal noise into the neural system. This noise is assumed to compensate for the reduced background neural activity in ADHD related to their hypofunctioning dopaminergic system [38, 39]. Soderlund et al. [22] propose that the required level of extra-task stimulation (noise) depends on dopamine functioning so that participants with low dopamine levels (such as children with ADHD) require more noise to reach optimal cognitive performance in comparison with typically developing children (TDC).

MBA model is corroborated by three studies using a long-term memory task [22, 26, 40], which indicate that an optimal level of noise for inattentive children has detrimental consequences for TDC. Helps et al. [26] also showed benefits from white noise in a Go/Nogo task in children who were considered as “sub-attentive” by their teachers, while performance in “super-attentive” children worsened. The benefits concerned omission errors, which were significantly reduced in the sub-attentive group (in the white noise exposure condition), while there was no effect on commission errors for any group. However, a direct link between dopaminergic functioning and the beneficial effect of white noise on cognition has not yet been clearly demonstrated [26, 38]. In a study with a rat model of ADHD [41], white noise exposure did not increase dopamine levels, and noise benefit could be found even in dopamine-lesioned rats. In humans, spontaneous eye-blink rate, a marker of dopamine functioning in the striatum [42, 43], might help to further investigate this relationship.

To our knowledge, the potential effect of a white noise exposure on a visual Go/Nogo task has not been previously examined in children with ADHD, nor at behavioral or at neurophysiological levels. The P300 is an event-related potential (ERP) component that allows different processes (see below) to be studied and is often used as an interest marker in ADHD [44–47]. ERP studies in ADHD using Go/Nogo tasks have generally shown reductions in P300 amplitudes at centro-parietal sites in children with ADHD when compared to TDC (in Woltering et al. [48]), though this may not always be the case (see [49, 50]). This attenuation of P300 amplitudes in individuals with ADHD may suggest that less attentional resources are allocated to inhibitory control and related evaluative processes [48].

In this study, the first objective was to compare noise benefit between children with ADHD and TDC in a visual cued Go/Nogo task using both behavioral and neurophysiological measures. This task, in which a non-informative cue precedes each Go or Nogo trial, enables the separate examination of P300 evoked by: (1) preparation (Cue P300); (2) inhibition (Nogo P300); (3) attention and orienting processes (Go P300; [51]). The Cued Go/Nogo was chosen because the high target/non-target ratio in this task is adapted to rapidly obtain the minimum of 36 artifact-free trials (by condition) required for measuring a P300 [52]. Moreover, white noise was already found to be beneficial in a study using Go/Nogo in children considered “sub-attentive” but without an ADHD diagnosis [26]. The Go/Nogo task is also one of the most frequent inhibition tasks used in ADHD [53]. The second objective was to investigate potential correlations between noise benefit and individual neuropsychological profiles. For that purpose, we proposed a new marker of performance, the “Noise Benefit Index” (NBI), which allowed us to consider whether or not participants had benefited from noise (present during the task), regardless of their group categorization (ADHD or TDC). The third objective was to investigate whether this NBI has a neurophysiological impact, i.e. whether differences in the P300 component can be observed between “noise-beneficiaries” (subjects who benefit from noise) and “noise non-beneficiaries” (subjects who do not). Finally, the fourth objective, was to measure spontaneous eye-blink rates to test the MBA model assumption that white noise would increase arousal through dopaminergic system modulation [38]. We hypothesized that (1) neuropsychological and neurophysiological differences would be observed between children with ADHD and TDC submitted to the visual cued Go/Nogo paradigm. We expected to observe more omissions, more impulsive errors, and slower and more variable RTs in the ADHD group but only in the no noise condition. In electrophysiological data, we expected to observe attenuated P300 amplitudes in children with ADHD compared to TDC in the no-noise condition. (2) We expected to observe increased P300 mean amplitude in the noise condition for noise beneficiaries and correlations between noise benefit and neuropsychological (and clinical) markers of attention and inhibition. (3) We expected a significant relationship between ADHD categorization and noise benefit categorization. (4) We hypothesized that children with ADHD would exhibit different eye-blink rates than TDC (in a no-noise condition) but that this difference would be reduced during white noise exposure.

## Methods

### Participants

Children with ADHD were recruited and assessed according to DSM IV-TR criteria [54] by a multidisciplinary team including pediatric neurologists and neuropsychologists in local university hospitals. If the child was regularly treated by methylphenidate, medication was stopped 48 h before testing. Exclusion criteria were a seizure disorder, IQ below 80, being in a specialized school, psychiatric comorbidities (assessed through the CBCL questionnaire; listed in Table 1), non-corrected sensory deficits and pharmacological treatment (other than methylphenidate) that could interfere with behavioral performance and/or with neurophysiological results. Children who have had otitis or other ear problems had an audiometry to ensure they had normal hearing.

**Table 1:** Means, standard deviations and group comparison for estimated IQ, age and parent-rated CBCL T-scores

Measure	Group				*t* test and ANOVA	
	TDCN = 17		ADHDN = 13		*t*	p
	Mean	SD	Mean	SD		
Estimated IQ	111.2	6.8	102.9	9.9	−2.72	.01*
Age	9.2	1.3	8.5	1.2	−1.66	.11
CBCL^a^						p^b^
Affective problems^a^	56.3	6.8	62.8	7.0	2.39	.14
Anxiety problems^a^	58.1	7.1	59.6	7.9	.53	.99
Somatic problems^a^	56.8	5.6	55.9	6.9	−0.36	1
ADHD problems^a^	53.2	5.5	64.8	7.6	4.51	<.01*
Oppositional defiant problems^a^	56.9	7.7	60.3	9.5	1.00	.99
Conduct problems^a^	58.4	8.9	62.7	9.5	1.18	.95

* p value indicating significant difference between groups; overall α = .05

^a^ Child behavior checklist; T-scores

^b^ p values below are corrected for multiple comparisons (Bonferroni correction)

At first, 36 children (7–12 years old) were recruited for the study. Three children were excluded because of a too poor signal-to-noise ratio after EEG qualitative observation. Two children were excluded because their estimated IQ was below 80, and one child asked to stop the experiment because it was too long for him. The two groups (ADHD and TDC) consisted then of 13 children with ADHD (5 girls; mean age = 9.2; SD = 1.3) and 17 TDC (9 girls; mean age = 8.5; SD = 1.2). TDC were recruited from primary schools.

In addition to the neuropsychological assessment (see 2.1.1.), each child performed subtests of the Wechsler Intelligence Scale for Children (WISC-IV: Wechsler, 2005). Estimated IQ was computed based on two perceptual processing subtests (picture concepts, matrix reasoning) and two verbal comprehension subtests (similarities, vocabulary) of the WISC-IV. Intellectual assessment was aimed at excluding children who presented an intellectual weakness but was not used for group comparison as it is known to be influenced by attentional and executive factors [55, 56]. Parents were asked to fill in the Child Behavior Checklist–CBCL [57]. Results from CBCL and IQ testing are shown in Table 1. As gender influences symptom expression and cognitive profile in ADHD [58], we performed a *Chi* square test of independence to assess whether the gender ratio was different between the groups. It showed that the gender ratio was not statistically different between the groups [*Chi* square (1) = 1.23, p = .27].

Informed consents were obtained from all subjects and from their parents with the prior approval of the Ethics Committee of the Queen Fabiola Children’s University Hospital (ULB, Belgium), of the Erasme Hospital (ULB, Belgium) and of the Faculty of Psychology and Education (ULB, Belgium).

### Material and procedure

Data was acquired during two separate sessions: a neuropsychological assessment and an experimental session.

#### Neuropsychological assessment

During this session, we used the computerized TAP battery [59, 60] and a Counting Stroop task [61] to assess different components of attention and executive functions. The TAP battery allows assessing several attentional and executive processes and is well normed (n > 500), both for children (from 6 years old) and adults, which allows using the same battery in children and adult studies. It has been shown to be an effective instrument to investigate both cognitive functions in ADHD (in children and adults) [60] and treatment efficacy in ADHD [62]. Attentional abilities were assessed using the Alertness subtest of the TAP. Alertness included a simple reaction time (tonic alertness) and an auditory-cued reaction time task (phasic alertness). The tonic condition represents a good measure of intrinsic alertness and the phasic alertness is used to evaluate the effect of a warning cue during attention tasks. Inhibition was evaluated using the Go/Nogo subtest of the TAP and a Counting Stroop task [61, 63]. The Go/Nogo task requires either a button press response (Go) or the inhibition of a response (Nogo), depending on the stimuli presented (the “go” is represented by an “×” and the “nogo” by a “+”). The Counting Stroop task included three conditions: counting, reading and interference. Items were presented on a computer screen in 10 lines, presented one at time, with 10 stimuli per line (squares with numbers or dots). In the counting condition, children had to report as fast as possible the number of dots within each square. In the reading condition, they had to read the number written within each square. In the interference condition, they had to report how many numbers were written within each square, while avoiding reading the number itself.

IQ testing as well as the completion of the CBCL questionnaire and the informed consent was performed during this session.

#### Experimental session

**Fig. 1:**
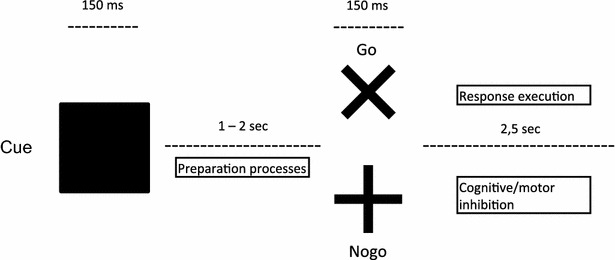
Illustration of the visual cued Go/Nogo task with stimuli, ISI and stimulus-related processes

In the visual cued Go/Nogo task, the child was submitted to three kinds of 3 × 3 cm stimuli briefly displayed (150 ms), in black, one by one on a grey background. A square (the warning stimulus hereafter called the cue) always preceded Go (“×”) or Nogo (“+”) stimuli. Go and Nogo stimuli each had a 50 % of probability of following a cue and were pseudo-randomly displayed (see Fig. 1). The inter-stimulus interval (ISI) between the cue and the following stimulus was varied randomly (1–2 s, mean = 1.5 s) while the ISI between Go or Nogo stimuli and the following cue was constant (2.5 s). The task was divided into two different blocks, each lasting 4 min 20 s. In each block, 60 cues were presented, 30 Go and 30 Nogo stimuli. Subjects had to perform each block twice, once with and once without white noise. There were thus altogether four blocks per participant. The order of the blocks and conditions (noise or no-noise exposure) was counterbalanced. Noise was delivered binaurally at 77 dB SPL with Etymotic earphones (model ER-3A) connected through a 25 cm long silicon tube ending in a hollowed foam cylinder inserted into the entrance of the ear canals.

The children were asked to press a button as fast as possible each time a Go stimulus was displayed and had to inhibit pressing when a Nogo was presented. We explained them that the square was supposed to help them prepare for the following stimulus presentation. Both speed and accuracy were encouraged.

For the sake of EEG recordings, the children sat in a sound attenuated room in a comfortable resting chair with headrest. Distance from the 17″ computer monitor was 120 cm.

### Electrophysiological recording

Brain electrical activity was recorded with an ASA EEG/ERP system (ANT software, The Netherlands) from 14 channels (Fz, F3, F4, Cz, C3, C4, Pz, P3, P4, Oz, O3, O4 and M1–M2 for the left and right mastoids), embedded in a waveguard cap (10–20 system) and all referred to the mean of the two mastoids. Horizontal and vertical eye movements were monitored using two bipolar recordings: one between each outer eye canthus and one between a supraorbital electrode and an electrode positioned just below the lower eyelid on the left side. The ground was placed on the left wrist. All impedances were kept below 10 kΩ. After amplification (×20) and online filtering (0.1–100 Hz, as recommended in Duncan et al. [52]), the input signals were digitized with a sampling rate of 512 Hz and stored on the computer disk for off-line averaging.

After the experimental task, children were asked to keep their eyes open to record eye-blinking rate, during two blocks of 2 min, one with and one without white noise exposure (in counterbalanced order).

### Data analysis

#### Neuropsychological assessment, IQ testing and CBCL

Independent samples t tests were used to assess group differences (ADHD vs. TDC) with regard to IQ, CBCL questionnaire as well as scores from the TAP and from the Counting Stroop.

In the TAP subtests, subject’s median RT was chosen as a measure of response latencies because it is less sensitive than the mean to the enhanced intra-individual variability in response time usually observed in the ADHD population [12]. Another dependent variable, estimating the intra-individual variability, was the coefficient of variation (CV) of reaction times [64], a normalized measure of dispersion, defined as the ratio of the standard deviation (σ) to the mean (μ): CV = σ/µ. The CV is useful because the standard deviation of data must be understood in the context of the mean of the data [65]. We also used hits, anticipations (in the phasic alert part of the Alertness part) and errors (in the Go/Nogo test) as measures of impulsivity [59].

Two variables were computed to investigate the classical interference effect in the Counting Stroop, i.e. the difference scores (e.g., [66]) between counting and interference conditions for total time (“time interference index”) and for total number of non-corrected errors (“errors interference index”).

#### Experimental task

Dependent variables were omissions (no responses to “Go” trials), false alarms (“FA cue”, pushing when the cue is shown and “FA Nogo”, pushing when “+” is shown), RTs and the RT variability.

#### Group assignation

Subjects were assigned to two kinds of groups. First, they were assigned according to their diagnosis status: “ADHD” or “TDC”. Second, they were assigned according to their benefit from noise or not during the task. Therefore, we computed a NBI that calculated the difference of omissions, for each subject, in noise vs. no-noise conditions (i.e. the percentage of hits in the noise condition minus the percentage of hits in the no-noise condition). Children with a higher NBI index (i.e. more hits/less omissions in the noise condition) were grouped as “noise beneficiaries” (n = 12) and the others (same or less hits/more omissions in the noise condition) were assigned to the “non-beneficiaries” group (n = 18). Mean and standard deviation (SD) for the NBI scores in each group are: ADHD (mean = 9.11, SD = 22.24); TDC (mean = −2.80, SD = 7.20), noise beneficiaries (mean = 15.20; SD = 19.71); noise non-beneficiaries (mean = −6.20; SD = 3.71).

A median split, another possible way to create two groups regarding noise benefit, might have assigned participants who made more omissions in the noise condition to the “noise beneficiaries” group. Therefore, the former method, although not perfect, was considered more appropriate. As gender influences symptom expression and the cognitive profile [58], we performed a *Chi* square test of independence to assess whether the gender ratio was different between the groups (“noise-beneficiaries” and “non-beneficiaries”). It showed that the gender ratio was not statistically different between the groups [*Chi* square (1) = .55, p = .46]. Age did not statistically differ between these groups [t(28) = −1.72; p = .10].

For all dependent variables, two mixed ANOVA’s were performed with between Group factors: (1) ADHD vs. TDC and (2) “noise beneficiaries” vs. “non-beneficiaries”. Within-subjects factors were block (first vs. second block) and condition (noise vs. no-noise).

*Chi* square test of independence was applied to examine the potential relation between ADHD and benefiting from noise. Aside from the Chi square test, an independent samples *t* test was used to assess the difference in the mean of the NBI score between ADHD and TDC groups. Pearson correlations were used to examine relationships between the NBI and neuropsychological scores.

#### Neurophysiological measures

Continuous EEG was segmented in 1200 ms epochs including a 200 ms pre-stimulus onset baseline. Averaged waveforms were computed for each subject and then across groups for each of the following trials: “Go” (“×”), only when the subject had responded, “Nogo” (“+”), only when the child did not respond and “cue” (square).

Blinks were corrected with the SOBI algorithm [67] to avoid rejecting too many epochs during averaging. By doing so, we kept at least 90 % of the epochs for each participant after averaging (with a rejection criterion at ±100 µV, as recommended by Duncan et al., [52]). Data were baseline corrected before statistical analysis. Mean amplitudes were individually identified by group, for each type of P300 (Cue, Go, Nogo) and at Cz and Pz. They were computed in a 300 ms temporal window centered on the most positive point visually inspected (on the grand average) for each of the three kinds of trials (Cue P300, Go P300 and Nogo P300), for each group, for each condition (noise and no-noise) and for each electrode (Cz and Pz).

For each “Type of P300” (Cue P300, Go P300 and Nogo P300), two mixed ANOVA’s were performed for mean amplitude with Groups: (1) ADHD vs. TDC and (2) “noise beneficiaries” vs. “noise non-beneficiaries” as between-subjects factors. “Site” (Cz, Pz) and condition (noise vs. no noise) were within-subject factors.

#### Eye-blink rates

Spontaneous eye-blink rates (after the task) were analyzed with Matlab 2012. A free Matlab script, “peakdet” ([68] cited in [69]), allowed us to count eye-blinks for each subject from their EEG recording.

We chose to register spontaneous eye-blinks after the main experiment (and not during) because our goal was to make an indirect inference between dopaminergic functioning and white noise. While eye-blinks during a task seem to reflect the transition of activation between different neural networks [70], spontaneous eye-blink rates reflect a more “natural-state” of dopaminergic functioning. The latter, therefore, was chosen because it was more related to our original hypotheses. Moreover, children were explicitly asked not to blink too much during the task. This could have biased our observations, contrary to the resting state condition (without such instruction).

A repeated measures ANOVA was performed for eye-blink rates with Groups: (ADHD vs. TDC) as between subject factor and condition (noise vs. no noise) as within-subjects factor.

All statistical analyses were performed using “Statistica 8.0”. When necessary, post hoc Tukey tests were applied.

## Results

### IQ testing, CBCL questionnaire and neuropsychological assessment

As illustrated in Table 1, TDC (IQ = 111.2 ± 6.8) performed higher than children with ADHD (IQ = 102.9 ± 9.9) on estimated IQ measures [t(28) = −2.72; p = .01]. CBCL T-scores were significantly different between groups for the ADHD subscale only [t(28) = 4.51; p < .01].

In the Alertness TAP subtest, analyses disclosed larger mean median RTs in ADHD than in the TDC group in tonic [t(28) = 2.18; p = .04] and phasic alertness condition [t(28) = 2.24; p = .03]. Coefficient of variation was also higher in ADHD for tonic [t(28) = 2.42; p = .02] and phasic alertness [t(28) = 3.31; p < .001]. ADHD children had less correct responses [t(28) = −2.93; p = .01] and made more anticipations [t(28) = 2.89; p = .01] than the TDC group.

In the Go/Nogo TAP subtest, coefficient of variation [t(28) = 2.21; p = .03] and the number of errors [t(28) = 2.48; p = .02] were larger in the ADHD than in the TDC group.

In the Counting Stroop task, analyses disclosed significantly larger time interference [t(28) = 4.46; p < .001] and errors difference indices [t(28) = 2.04; p = .05] in ADHD than in TDC.

All results of the TAP tests and the Counting Stroop are shown in Table 2.

**Table 2:** Means, standard deviations and group comparison for TAP (tonic and phasic alert, Go/Nogo) and Counting Stroop tests

Measures	Group				*t* test	
	TDC N = 17		ADHD N = 13		*t* values	p
	Mean	SD	Mean	SD		
Alertness (tonic)^a^						
Median	305.35	71.12	421.00	203.96	2.18	.04*
CV	0.22	0.08	0.32	0.14	2.42	.02*
Hits	40.00	0.00	39.62	0.96	−1.66	.11
Anticipations	0.00	0.00	0.00	0.00	–	–
Alertness (phasic)^b^						
Median	281.94	55.20	353.38	115.81	2.24	.03*
CV	0.17	0.05	0.31	0.17	3.31	.00*
Hits	39.29	1.82	35.08	5.59	−2.93	.01*
Omissions	0.00	0.00	0.54	1.45	1.54	.13
Anticipations	5.00	4.55	12.08	8.69	2.89	.01*
Go/Nogo						
Median	521.88	79.15	584.83	92.01	1.97	.06
CV	0.24	0.06	0.29	0.05	2.21	.03*
Errors	2.59	2.12	5.23	3.68	2.48	.02*
Omission	0.71	1.21	3.15	5.16	1.90	.07
Stroop^c^						
Time interf. index	39.59	16.41	72.46	23.95	4.46	<.001*
Errors interf. index	0.35	0.70	2.08	3.40	2.04	.05*

^a^ Scores of tonic alert in alertness task

^b^ Scores of phasic alert in alertness task

^c^ Scores representing the difference of performance between counting and interference conditions: *Time interf. index.* difference of total times; *Errors interf. index* difference of non-corrected errors

* p value indicating significant difference between groups; overall α = .0

### Cued Go/Nogo experimental task results

Relevant behavioral and electrophysiological data are presented according to the comparison between ADHD and TDC groups in Section 1 and according to the comparison between “noise-beneficiaries” and “non-beneficiaries” groups in Section 2. Block factor was included in each behavioral analysis but will not be presented, as the results were not relevant in this context.

#### Section 1: ADHD vs. TDC

Behavioral measures

*Omissions*

**Fig. 2:**
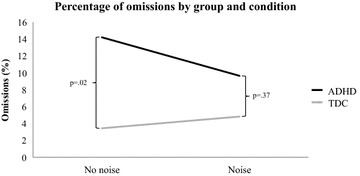
Percentage of omissions by Group and Condition. This figure indicates a significant difference between the groups in the no-noise condition only, ADHD making more omission in that condition than TDC

Children with ADHD made more omission errors than TDC [F(1,28) = 5.69, p = .02]. There was no main effect of Condition [F(1,28) = 1.21, p = .28], but a significant Group*Condition interaction (see Fig. 2) [F(1,28) = 4.32, p = .04] indicated that children with ADHD made more omissions than TDC in the no-noise condition only (p = .02). There was no other interaction (all Fs < 2.23).

*RTs and RT variability*

Children with ADHD were slower [F(1,28) = 9.98, p = .004] an more variable [F(1,28) = 9.98, p = .04] than TDC. All other factors did not reach significance and did not interact with each other (all Fs < 1).

*FA cue*

Children with ADHD committed more FA cues than TDC [F(1,28) = 4.14, p = .051]. No other factor reached significance and there was no interaction (all Fs < 2.96).

*FA nogo*

No factor reached significance and there was no interaction (all Fs < 2.04).

Electrophysiological measures

*Cue P300*

Cue P300 amplitudes were similar regardless of the Group, Condition or Site and there was no interaction (all Fs < 2.88).

*Go P300*

Go P300 amplitudes were similar regardless of the Group, Condition or Site and there was no interaction (all Fs < 1).

*Nogo P300*

There was a Group*Site interaction [F(1,28) = 32.48, p < .001] and a three-way interaction Group*Condition*Site [F(1,28) = 5.04, p = .03], indicating that ADHD had a significant higher Nogo P300 than TDC but only at Pz in the noise condition (p = .05). No other factor reached significance and there was no interaction (all Fs < 3.75).

#### Section 2: Noise beneficiaries vs. noise non-beneficiaries

In this section, children who benefited from noise (noise-beneficiaries) were compared to those who did not (non-beneficiaries), independently of their diagnostic status. Given that we split our groups according to the fact that children benefited from noise on omissions errors, analyzing behavioral effects on these omissions would be redundant and was not included. Here we present analyses on electrophysiological because no relevant behavioral analysis reached significance. The clinical status * noise benefit * block * condition four-way mixed ANOVA would have allowed quantifying the interaction effect of clinical status and noise benefit on our dependent variables. However, we did not follow this approach because the two types of group assignment yielded an imbalanced design with cells with low sample sizes (see Table 4).

Electrophysiological measures

*Go P300*

**Fig. 3:**
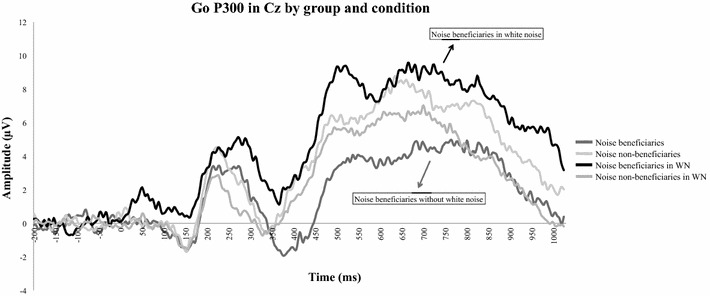
Grand averages of the Go P300 at Cz by Group and Condition. Positivity is plotted up. *WN* white noise. Mean latency of the most positive points on the different grand averages are: noise beneficiaries = 764 ms; noise non-beneficiaries = 634 ms; noise beneficiaries in WN = 664 ms; noise non-beneficiaries in WN = 700 ms

There was a three-way interaction Group*Condition*Site [F(1,28) = 9.62, p = .005] indicating, as illustrated in Fig. 3, that the amplitude of the Go P300 increased marginally for noise beneficiaries at Cz in the noise relative to the no-noise condition (p = .06). All other factors did not reach significance and did not interact with each other (all Fs < 1.38).

There was no relevant result for Group, Condition or Site factors as regards Cue and Nogo P300.

### The Noise Benefit Index (NBI) and its relationship to ADHD and cognitive functions

All significant correlations between NBI and neuropsychological tests (TAP and Counting Stroop) are presented in Table 3. Both markers of inattention (CV and hits from Phasic Alert test) and of motor and cognitive inhibition (respectively, anticipation from the Phasic Alert and the errors interference index from Stroop test) exhibited correlations with the NBI.

**Table 3:** Significant correlation between NBI and neuropsychological tests

NBI^a^ correlations	r	p	p *corr* ^*b*^
Alertness (tonic)^c^			
CV	.62	<.01*	<.01*
Alertness (phasic)^d^			
Hits	−.50	<.01*	.02*
Anticipations	.45	.01*	.04*
Stroop^e^			
Errors interf. index	.69	<.01*	<.01*

^a^ *NBI* Noise Benefit Index

^b^ p value corrected for multiple comparison (for each cognitive function, p values were corrected according to the number of dependent variables)

^c^ Scores of tonic alert (alertness task)

^d^ Scores of phasic alert (alertness task)

^e^ *Errors interf. index* difference of non-corrected errors between scores in counting and interference conditions

* p value indicating significant difference between groups; α = .05

These correlations indicate a relationship between benefitting from noise and lower attention and inhibition (motor and cognitive) in the neuropsychological tests.

The Chi square test highlighted a significant relationship between the Group factor (ADHD or TDC) and benefitting from noise [Chi square (1) = 4.43 p = .035; see Table 4]. Children with ADHD had a significantly higher NBI score than TDC [t(28) = 2.08; p = .04].

**Table 4:** Repartition of each subject according to their categorization (ADHD or TDC) and to their benefit from noise during the visual cued Go/Nogo

Groups	Non-beneficiaries^a^	Noise-beneficiaries^b^
TDC	13	4
ADHD	5	8

^a^ *Non*-*beneficiaries* all subjects who did not benefit from noise

^b^ *Noise*-*beneficiaries* subjects who benefitted from noise

### Eye-blink rates

Children with ADHD made significantly more eye-blinks than TDC [F(1,28) = 5.94, p = .02]. There was no other significant effect or interaction (all Fs < 1.09).

## Discussion

In this study, we first aimed to evaluate the potential cognitive benefit of white noise exposure during a visual cued Go/Nogo task in children with ADHD and TDC. The second objective was to examine, through the use of a new index (NBI), whether potential correlations could be observed between noise benefit in the visual cued Go/Nogo task and individual neuropsychological profiles. The third objective was to investigate whether this NBI had a neurophysiological correlate, i.e. whether differences in the P300 component can be observed between “noise-beneficiaries” and “non-beneficiaries”. The fourth objective was to discuss, through the use of spontaneous eye-blink rates, the Moderate Brain Arousal model according to which white noise modulates dopaminergic functioning [38].

Regarding participant characteristics’, ADHD and TDC differed in the CBCL only in the ADHD problems subscale, indicating a limited influence of comorbidities on results. Results from the attentional and executive assessment showed significant differences between children with ADHD and TDC. Children with ADHD were slower and had higher RT variability on all neuropsychological tasks (phasic and tonic alert, Go/Nogo and our experimental task), confirming previous observations [71–73]. They made more omissions (phasic alert and Go/Nogo) and showed more impulsivity, as indexed by anticipations, Go/Nogo and interference in the Stroop.

Similar results were obtained in the experimental task. Children with ADHD made more omissions, more impulsive errors (i.e. FA cue) and had slower and more variable RTs than TDC. However, exposure to white noise during the experimental task showed a positive impact on cognitive performance in children with ADHD. To the best of our knowledge, this is the first demonstration of a beneficial effect of white noise in the Go/Nogo paradigm in a study comparing children with and without ADHD. Positive effects of white noise have already been documented on memory [22] and on Go/Nogo performance [26], but this last study was conducted with children teacher-rated inattentive. Taken together, results from the present study are in accordance with the optimal stimulation theory and the MBA model, both suggesting that the addition of extra-task stimulation is likely to improve cognitive functioning in ADHD. Moreover, these results help to clarify what kind of improvement might be expected (at 77 dB). Indeed, improvement was limited to omissions only (as in [26]). RTs and RT variability, as well as the number of false alarms (i.e., FA cue) remained significantly higher in children with ADHD compared to TDC, independent of the condition. Consequently, the beneficial effect of white noise on cognition is not generalizable to all attentional and executive functions but seems, in this task, to modulate vigilance more specifically. No Block effect was observed, which might have shown a larger beneficial effect of white noise exposure during the second half of the task, namely a real benefit in the wake of fatigue. A longer task might disclose such an effect, and this should be considered in future studies.

Electrophysiological data showed a significant Group*Condition*Site interaction with a higher Nogo P300 amplitude in ADHD than in TDC at Pz in the noise condition. This result might indicate enhanced inhibitory processes in children with ADHD in the noise condition. However, behavioral results did not show a similar effect, as we did not observe a difference between groups according to noise exposure. Possibly, this Nogo P300 underlines a positive neurophysiological effect of white noise that is not observed at the performance level (see e.g. [74]). More sensitive inhibition tasks (such as the stop-signal task [75, 76]) could be used in future studies. Electrophysiological data did not show other relevant difference between children with ADHD and TDC, regardless of the condition (noise or no-noise) and the type of P300—Cue P300, Go P300 and Nogo P300—that are associated with behavioral processes (preparatory processes, attentional and inhibitory processes respectively [51]. This suggests that despite the same amount of attentional resources allocated to these different cognitive processes in both groups [48], children with ADHD had worse behavioral performance. This observation highlights the heterogeneity of findings according to ERP research in ADHD [50]. With regard to the literature [48] and our behavioral results, we anticipated both a reduced Cue and Go P300 mean amplitudes in children with ADHD compared to TDC. Other studies have also found absence of effect on P300 amplitude despite a significant decline in performance in ADHD group [44, 77]. We suggest that this absence of neurophysiological difference can be explained by our non-comorbid group of children with ADHD. Indeed, Yoon et al. [78] showed that ADHD-comorbid (with ODD or CD) but not ADHD-pure children displayed significant P300 amplitude reduction compared to TDC.

When groups were assigned according to their noise benefit (noise-beneficiaries and non-beneficiaries), we demonstrated a significant relationship between group classification (ADHD or TDC) and benefiting (or not) from noise. In addition, we found significant correlations between benefitting from noise and markers of vigilance (RT variability, omissions) and motor/cognitive inhibition (anticipation errors and interference errors) in Stroop and TAP tasks (all correlations = p ≤ .01). These markers have been identified as core cognitive symptoms in ADHD [9].

Furthermore, noise-beneficiaries had a marginally larger Go P300 mean amplitude in the noise condition compared to the no-noise condition. This was not found in participants who didn’t benefit from noise. Interestingly, white noise modulates only, at an electrophysiological level, the Go P300 mean amplitude (for noise-beneficiaries), which is associated with attentional processes (like vigilance); and white noise modulates only vigilance at a behavioral level (and not impulsive errors, RTs or RT variability) in children with ADHD. We propose two possible complementary explanations: (1) White noise, perceived as a potential distractor, could make noise beneficiaries (prone to distraction regarding their correlations with markers of vigilance) gathering up more attentional resources not to be distracted. (2) The perceptual load hypothesis [79] proposes that increasing perceptual load (adding “task irrelevant-distractors”) reduces, or even eliminates, any distractor interference effect. Lavie [79] suggests that small increases in perceptual load may be beneficial for populations that are prone to distraction (e.g. children with ADHD). In our study, white noise, increasing general perceptual load, indeed reduces the inattention/improves vigilance (expressed in numbers of omissions) of children who are more easily distracted.

Finally, noise benefit on cognitive functioning supports the MBA model. However, it is unclear whether this noise improves performance through dopamine system modulation, as suggested by Sikstrom, Soderlund [38]. We addressed this issue by analyzing the spontaneous eye-blink rate, an indirect marker of dopamine functioning in the striatum [42, 43, 80, 81]. Spontaneous eye-blink rate measures showed that children with ADHD made more eye-blinks than TDC, which supports the relation between ADHD and dopaminergic system and could, therefore, be considered as a potential measure for future studies. However, noise did not significantly modulate eye-blink rate, which does not support the MBA model assumption. Yet, recent papers presented direct evidence that the central dopaminergic activity is involved in the modulation of P300 parameters [82, 83]. Given our finding of a modulation of the Go P300 by noise (for noise beneficiaries), the MBA model hypothesis is not to be ruled out but rather reviewed.

## Limitations of the study

Some limitations of our study have to be mentioned including, first, the small number of participants. Second (probably related to the first), the three-way interaction showing a larger Go P300 for the noise beneficiaries in the noise condition was only a trend. Third, in ERP studies on cognition, it is recommended to use two-year groupings over the age of 8 years because of significant ERP changes over a short time period [84]. Due to the difficulty of recruiting (with respect to the exclusion criteria and the characteristics of the experiment) and the small number of subject, the groups would be too small to consider this recommendation. Future studies with larger samples are needed to further investigate these original and new findings. Fourth, our NBI was calculated according to the number of omissions to create groups, while other factors, such as RT-variability, are also considered as pathways that contribute to distinguishing children with and without ADHD [85]. From our point of view, choosing another component (such as RT-variability) remains interesting but omission was the only one, in this present study, that seemed to be modulated by noise exposure. Therefore, it was considered to be more relevant.

## Conclusion and future direction

The finding of a white noise benefit in children with ADHD during a Go/Nogo task validates the optimal stimulation theory. However, the benefits of white noise are not to be generalized to all analyzed functions within this task and seem more specifically associated with vigilance improvement. The NBI allowed us to characterize a neuropsychological profile, related to that of ADHD, positively sensitive to noise at both behavioral and electrophysiological levels. We also provide neurophysiological correlates of noise benefit. The eye-blink rate investigation distinguishes ADHD between TDC groups, but was not sensitive to white noise, questioning the influence on dopaminergic functioning suggested by the MBA model. With regard to the literature, the type of extra-task stimulation has a different impact on cognitive functioning according to task requirements and interpersonal differences [38, 86]. Future research should manipulate these different parameters to better understand which stimulation improves/modulates the different executive or attentional function. The present study should be conducted in adults with and without ADHD to see if development is a crucial factor to take into account. It would be of interest to measure eye-blink rates during the task in a future study, and avoiding asking children explicitly to not blink during the test. Finally, while this study highlighted the potential benefit of adding stimulation in the environment, it is time to consider children’s on-task behavior in a more ecologic environment (e.g., a virtual classroom) to fully understand what situational factors help moderating difficulties for children with ADHD [87, 88].

## Footnotes

1 White noise is a continuous random signal-sound from 20 to 20,000 Hz [23].
